# Post acute CoViD-19 syndrome (PACS) - Long CoViD

**DOI:** 10.6026/97320630018908

**Published:** 2022-10-31

**Authors:** Francesco Chiappelli, Lily Fotovat

**Affiliations:** 1 Center for the Health Sciences, UCLA, Los Angeles, USA; Dental Group of Sherman Oaks, CA 91403, USA

**Keywords:** Coronavirus (CoV), Middle-East Respiratory Syndrome (MERS), Severe Acute Respiratory Syndrome (SARS), Systemic Acute Respiratory Syndrome Corona virus2 (SARS-CoV2), World Health Organization (WHO), Center for Disease Control (CDC), Corona Virus Disease 2019 (CoViD-19), Angiotensin-converting enzyme (ACE), exhausted T cells (Tex), interleukin (IL), tumor necrosis factor (TNF), Post-COVID-related osteonecrosis of the jaw (PC-RONJ), Post Acute CoViD-19 Syndrome (PACS)

## Abstract

Patients sero-positive for the Systemic Acute Respiratory Syndrome Corona virus2 (SARS-CoV2) virus develop the Corona Virus Disease 2019 (CoViD-19). CoViD-19 may be asymptomatic in some individuals, proffer mild symptoms in other patients, and can be a serious and even lethal disease in a sub-group of the population. The variables that determine the severity of CoViD-19 have not been fully characterized. What is clear is that the patients who survive CoViD-19 return to a state of sero-negativity for SARS-CoV2 generally within 3-5 weeks. However, several cases of repeated infection have been reported, and a large proportion of CoViD-19-recovered patients manifest multi-system and multi-organ symptomatic pathologies several weeks-to-months after resuming sero-negativity for SARS-CoV2. This new pathological condition, originally termed Long Covid, is now recognized as the Post Acute CoViD-19 Syndrome (PACS). The original principal clusters of signs and symptoms of PACS: likelihood of relapse and reinfection, physical fatigue and cognitive slowdown, may actually be broadened to include immune deregulation, cardiovascular disease and coagulation abnormalities. The development and evaluation of new and improved clinical interventions for PACS are critical and timely.

## Background:

On 31 December 2019, the World Health Organization (WHO) was alerted to several cases of pneumonia in Wuhan City, Hubei Province of China. The causative pathogen was suspected to be a virus, but it did not match any other known virus. On 7 January 2020, Chinese authorities confirmed that they had identified the causative agent as a novel Coronavirus (CoV), the family that includes viruses known to cause Middle-East Respiratory Syndrome (MERS) and Severe Acute Respiratory Syndrome (SARS). The new CoV was named Novel Coronavirus (emerged late) 2019 (2019-nCoV). On 12 January 2020, Chinese scientists released the genetic sequence of 2019-nCoV. Shortly thereafter WHO and Center for Disease Control (CDC) established that 2019-nCoV, renamed as Systemic Acute Respiratory Syndrome Corona virus2 (SARS-CoV2) was responsible for Corona Virus Disease 2019 (CoViD-19) [1,2 &^3^].

By March of that same year, we argued that, since the Spike protein of SARS-CoV2 is a principal ligand to the ubiquitous Angiotensin-converting enzyme (ACE) 2 receptor, it was likely and even possible that SARS-CoV2 infection might target multiple tissues and organs, and that CoVid-19 may manifest as a complex multi-dimensional pathology. We discussed the likelihood that SARS-CoV2 may seriously impair anti-viral immune surveillance by enhancing that component of cellular immunity manifested as 'exhausted T cells' (Tex). Tex are primarily of the CD8+ population and emerge in chronic high-grade viral infection, such as what is observed in CoViD-19. We noted that Tex express certain specific markers, including CD279 (programmed cell death marker-1, PD-1) and CD366 (T cell immunoglobulin & mucin domain-3, Tim-3), and either produce or are modulated by certain cytokines, including interleukin (IL)-10. Tex are characterized by progressive loss of effector functions, high and sustained inhibitory receptor expression, metabolic deregulation, poor memory and homeostatic self-renewal, and distinct transcriptional and epigenetic programs. PD-1 (CD279) and Tim-3 (CD366) expression are critical checkpoints for Tex, as are several other biomarkers. Indeed, in vitro IL-10 blockade, or administration of IL-2, which overrides PD-1 inhibitory signaling, can reverse T cell exhaustion in experimental settings. In brief, the Tex sub-population is heterogeneous and includes 2 principal subsets: a progenitor and a terminal subset with unique characteristics and responses to checkpoint blockade. In brief, at the initial and intermediate stage, Texin are CD8+CD279low/medium, retain quasi-normal mitochondrial spare respiratory capacity, and are generally responsive to PD-1 blockade. At the more advanced stage, Texad are CD8+CD279 high, have lower mitochondrial respiratory capacity, and are terminally exhausted and largely unresponsive to PD-1 checkpoint blockade.

Broadly speaking, the expression of CD366 (Tim-3) in Tex parallels that of CD279 (PD-1), as well as the production of IL-10, tumor necrosis factor (TNF)-α and other cytokines that modulate the expression of either CD279 or CD366, or both, and mediate apoptotic T cytopeania. We predicted that SARS-CoV2 infection could gain ground not only by virtue of the properties of the virus (i.e., binding, replication, shedding), but also by blunting anti-viral immunity and causing severe immunopathology. Although we did not use the term "cytokine storm", which soon thereafter became a common phrase to describe the deregulated rise of a variety of pro-inflammatory, TH1 and TH2 cytokines as one primary feature of the CoViD-19 pathological profile, we did mention the triad IL-1β, IL-6, and TNF-α, as key inflammatory markers in SARS-CoV2 pathology [2].

In April 2020, we forewarned that, since ACE mediates neuro inflammation, neurodegeneration and neurotoxicity which are responsible for several CNS disorders, and since ACE2 counteracts the damaging effects of ACE on central nervous system neurons, it was probable and even likely that SARS-CoV-2 could directly access and infect the central nervous system via the circulation or via cranial nerve I, the olfactory bulb and possibly the nano-oral mucosa of the soft palate. In that manner, inactivation of ACE2 following binding of the SARS-CoV-2 Spike protein to ACE2-R in situ , we defended, could be expected to blunt the ACE2-moderating effects upon ACE neurotoxicity and neurodegeneration. We predicted that CoViD-19 may have clinically important central nervous system pathology, with possible cognitive and psycho-emotional derangements, and we proposed the likely biological mechanism for those outcomes [3].

By May 2020, it was clear that CoViD-19 was a complex disease that manifested a wide spectrum of signs and symptoms, from immune deregulation and severe unregulated cytokine storm, to altered coagulation parameter, leading to serious micro-thrombi, to acute respiratory distress syndrome with severe cough, dyspnea and pneumonia, severe gastrointestinal disorder with diarrhea, liver injury, confusion and neuro-cognitive issues, in addition to fever, chills, fatigue, generalized myalgia, malaise, and drowsiness. To be clear, it was becoming increasing evident that SARS-CoV-2 infection results in a wide variety of symptoms and pathologies, which ought to be best subsumed under the term "syndrome" then "disease". We proposed that the condition displays a multi-dimensional spectrum of symptoms reminiscent more of a syndrome, than a specific disease, and that, therefore, the natural history of this pathology may be better recognized as Corona Virus Syndrome 2019 (CoViS-19) [4].

To be sure, fundamental and clinical research has widely confirmed our hypotheses and expanded the knowledge-base about the virology and immunopathology of SARS-CoV-2 [5, 6, 7 &^8^], the immune response to infection and reinfection [8, 9 & 10], and the multi-organ and multi-system nature of CoViD-19, from the neurological and central nervous system pathologies [11, 12, 13 & 14], respiratory [15,16], cardiovascular [17, 18 & 19], gastrointestinal [20, 21 &^22^], endothelial and coagulation [23,24], with micro-thrombi complications, from deep vain thrombosis to pulmonary embolism [25, 26 and 27]: a complex set of multiple pathologies [28,29]. In all, five principal clusters of CoViD-19 pathology have been identified statistically to date [30].

Between January 2020 and October 2022, SARS-CoV-2 infection fast evolved into a global pandemic and a serious international public health threat. It has infected, according to Worldometer statistics (www.worldometers.info) well over 622 million men, women and children across all the countries of the world, and has led to over 6.5 million deaths. It also has resulted in over 602 million people who recovered from CoViD-19: some were sero-positive for SARS-CoV2 but asymptomatic, others had symptoms of varying degrees of severity. Several patients had rebound infections. All in all, public health specialist count 6-8distinct waves of infection on average across different countries during the previous 30 months. The nature of Corona viruses, being positive-sense single stranded RNA, produce a wide propensity for genomic variations and mutations; and indeed, SARS-CoV2 has produced a large number of variants and sub-variants of diverse strength, infectivity, and pathogenicity, compared to the original strain initially isolated and characterized in January 2020. These inherent properties of the virus blunt the fundamental virus interference processes, which can often be expected to mitigate viral epidemics and pandemics [31].

It is now widely reported that a sizable group of patients who have recovered from an initial bout with CoViD-19 experience what has been loosely termed Post-Covid conditions, or Long Covid, weeks or even months after returning to an SARS-CoV2 sero-negative state. They suffer from a variety of ongoing health problems, which range from fatigue to cognitive impairments, from gastrointestinal to cardiovascular problems, and from metabolic and immune imbalances to sustained coagulopathy. In general, these patients 35 weeks and beyond following acute CoViD-19 (i.e., Long Covid) still report multi-system involvement and significant systemic, and neuro-cognitive symptom burden, and cannot return to previous levels of athleticism, work or regular lifestyle activities [32]. Oral manifestations in Long Covid are likely and even possible because of the suspected direct vulnerability of the oral mucosa to SARS-CoV2 infection, and of the high ACE-2 expression in oral epithelial cells and keratinocytes [33, 34 & 35]. Case in point, a growing number of osteonecrosis cases have been reported in Long Covid, to the extent that Post‐COVID‐related osteonecrosis of the jaw (PC‐RONJ) is now considered as a clinically relevant maxillofacial complication in Long Covid [36].

A large meta-analytical study, comprising a total of 1,680,003 hospitalized and non-hospitalized SARS-CoV2 sero-positive patients, established that a sizable number of people who recover from CoViD-19 manifest a significant symptomatic burden as early as one month, and as late as 4 months from being disease-free. Although discrete regional differences were observed: notably, in Asia, roughly 51% of the recovered patients exhibited Long Covid after one month, whereas roughly 31% of the patients in the US, and 44% of the patients across Europe. The global prevalence of Long Covid was computed to be 0.37 (i.e., 37%) (95% CI, .26-.49) at 30 days, 0.25 (95% CI, .15-.38) at 60 days, 0.32 (95% CI, .14-.57) at 60 days, and 0.49 (95% CI, .40-.59) at 120 days - meaning to say that between 30 and 90 days after resolution of CoViD-10 about one in three patients is at risk of exhibiting signs and symptoms of Long Covid, and that this proportion dramatically rises to one in two at 4 months [37].

In brief, and based on the statistics noted above, of the 602 million people who have recovered from CoViD-19, a very large number indeed, can be expected globally to develop or to exhibit multi-system manifestations of Long Covid, now recognized as the new pathological entity of the post-acute CoViD-19 syndrome (PACS) [38, 39, 40 & 41]. The five most common symptoms, that is to say the five principal symptomatic clusters of PACS are fatigue (58%), headache (44%), attention disorder (27%), hair loss (25%), and dyspnea (24%), in addition to the propensity of returning to a state of sero-positivity and developing CoViD-19 again [42]. Three additional pathological clusters may certainly be added soon to the symptomatology of PACS: immune deregulation (e.g., persistent inflammation, induced autoimmunity, cellular immune deregulation) [43, 44], cardiovascular disease and endotheliopathy and coagulopathy [45, 46].

## Conclusion:

The development and evaluation of new and improved clinical interventions for PACS are now critical and timely. One current approach included offering patients who were treated for CoViD-19 pneumonia a follow-up appointment [47]. Only patients with an RT–PCR confirmed CoViD-19 pneumonia were included in the cohort study. A standard dataset was collected for each patient, including demographics, details of acute CoViD-19 admission, treatment, and symptom burden follow-up. Approved questionnaires were used to quantify dyspnoea by means of the MRC dyspnea scale [48] as well as quality of life with the 5-level EuroQol-5 Dimension (EQ-5D-5L [49]). Other symptoms of Long Covid Pacs patients were assessed using a standardized in-house follow-up assessment designed to establish whether or not the patients were currently experiencing specific symptoms [47]. Data from the CoViD-19 Symptom Study App [50] identified self-reported fatigue as the most common complaint in a large group of Long Covid PACS patients. If these symptoms persisted for more than four months, the patients were directed to meet the National Institute for Health and CARE Excellence (NICE) diagnosis criteria for Chronic Fatigue Syndrome [47]. Patients reported post-exertional fatigue, cognitive difficulties, sleep disturbance, and chronic pain. A systematic review of exercise therapy for Chronic Fatigue Syndrome concluded that completion of exercise therapy improved sleep and physical function and decreased perception of fatigue in these patients [51]. The prevalence of Long Covid PACS symptoms is often reported to be not different among hospitalized and non-hospitalized CoViD-19 patients [51]. Therefore, to combat the lingering symptoms of Long Covid PACS, clinical trials [52] incorporate interviews, focus groups, data management and analysis, and a patient involvement statement. Patients-interviewees may invite to tell their stories to help identify the common underlying illnesses and emotional touch points in a typical PACS illness journey. It is noteworthy that participants generally report finding accessing care complex, challenging, and even exhausting [52]. In conclusion, greater understanding of the pathogenesis, risk factors, symptoms, and methods of treating Long Covid PACS must ensure access to care, reduce the burden of illness, take clinical responsibility, provide continuity of care, and further develop the knowledge base and clinical services. The development and evaluation of modes of rehabilitation targeted to this novel pathological syndrome will have address the five identified symptomatic clusters of this condition: dyspnea, fatigue, and musculoskeletal, cognitive, and mental health impairments [53], to which should be added immuno pathology and cytokine storm, coagulation abnormalities and cardiovascular disease.
